# Identification of Tumor Antigens and Immune Subtypes in Hepatocellular Carcinoma From a Multiomics Perspective

**DOI:** 10.1002/cnr2.70300

**Published:** 2025-09-15

**Authors:** Chunming Wang, Lei Cai, Qingyu Xie, Cheng Zhang, Xuefang Chen, Peng Cui, Guoqiang Wang, Shangli Cai, Yusheng Han, Kaihang Zhong, Guolin He, Shunjun Fu, Yuyan Xu, Junming He, Mingxin Pan

**Affiliations:** ^1^ General Surgery Center, Department of Hepatobiliary Surgery II, Guangdong Provincial Research Center for Artificial Organ and Tissue Engineering, Guangzhou Clinical Research and Transformation Center for Artificial Liver, Institute of Regenerative Medicine, Zhujiang Hospital Southern Medical University Guangzhou China; ^2^ Department of Hepatobiliary Surgery The Second Affiliated Hospital of Guangzhou University of Chinese Medicine Guangzhou China; ^3^ Burning Rock Biotech Guangzhou China

**Keywords:** hepatocellular cancer, immune subtype, multiomics, tumor antigens

## Abstract

**Background:**

Immunotherapies including immune checkpoint inhibitors and tumor antigen based vaccines have revolutionized cancer treatment. However, immune signatures of hepatocellular carcinoma (HCC) have not been thoroughly studied from a multiomics perspective.

**Aims:**

In this study, we aimed to identify the potential tumor antigens and immune subtyping for HCC using multiomics data.

**Methods and Results:**

The study included 159 HCC patients with genome, transcriptome, proteome, and phosphoproteome data. Two potential tumor antigens, ZNF831 and SYNE1, showed significant superior prognostic effects and positive correlations with antigen presenting cells, which provided promising candidates for the development of tumor antigen‐based mRNA vaccine. A multiomics clustering using the most variable tumor antigen genes of transcriptome, proteome, and phosphoproteome was performed, resulting in two HCC subtypes with distinct clinical and molecular features. In order to further explore the complex tumor microenvironment, we implemented an immune subtyping using 30 most important immune‐related features generated from the random forest algorithm. Four immune subtypes were constructed, which exhibited diverse molecular attributes including immune cell activities, angiogenesis, cell proliferation, and the expression of tumor antigen genes, immune checkpoints, and immunogenic cell death modulators.

**Conclusion:**

In summary, we found two potential tumor antigens, ZNF831 and SYNE1, and identified four immune subtypes of HCC with distinct molecular features. Our study provides novel insights into the development of cancer vaccine and precision medicine of immune oncology in HCC, which may benefit clinical practice in the future.

## 1 Introduction

Liver cancer is the sixth most common cancer with a rapidly increased incidence rate worldwide and ranks as the third leading cause of global cancer mortality, accounting for 905 667 new cases and 830 180 deaths in 2020 [1]. Hepatocellular carcinoma (HCC) is the main pathological subtype of liver cancer, representing about 90% of all primary liver malignancies [2]. The etiology of HCC varies from cirrhosis, the chronic infection of hepatitis B virus (HBV) and hepatitis C virus (HCV), alcohol abuse, nonalcoholic fatty liver disease (NAFLD), and metabolic syndrome [3]. The HCV‐related HCC accounts for the most cases in developed countries, such as the United States and Japan. On the contrary, about 85% of HCC patients in China are derived from the chronic infection of HBV [4]. The survival of patients with HCC is largely determined by tumor stage at diagnosis; however, in general, the 5‐year survival rate of patients with HCC is less than 20%, which has put the disease into one of the cancers with the worst prognosis [5].

In recent years, cancer immunotherapy has achieved revolutionary improvements in the treatment of many cancers, especially antibodies against cytotoxic T lymphocyte‐associated antigen‐4 (CTLA‐4), programmed death‐1 (PD‐1), and programmed death‐ligand 1 (PD‐L1), which have brought significant survival benefits [6, 7, 8, 9]. Besides these immune checkpoint (ICP) inhibitors, novel tumor antigens have also attracted increasing attention. Tumor‐specific antigens are generated from genetic variants in tumor cells, such as single nucleotide variants (SNVs), insertions or deletions (indels), alternative splicing, and gene rearrangement, which have the potential to produce mutated peptides [10, 11, 12]. Some tumor‐specific aberrant peptides can interact with major histocompatibility complex (MHC) molecules and be presented on the surface of a tumor cell [13, 14]. They are attractive targets for T cells to recognize cancer cells because of their selective expression in tumors, which can stimulate strong antitumor immunity [15, 16]. Nowadays, personalized vaccines based on tumor‐specific antigens have shed light on the widespread clinical applications in various tumor types. Rosenberg's team transferred CD4+ T helper 1 cells, which could recognize a mutation in ERBB2IP expressed by tumor cells from a patient with metastatic cholangiocarcinoma, resulting in tumor regression [17]. Wu et al. developed neoantigen‐based polyfunctional CD4+ and CD8+ T cells for six melanoma patients, and results showed that four of them were free of relapse 25 months after vaccination [18]. The combination of personalized tumor antigen vaccine and PD‐1 blockade is also being studied in patients with advanced melanoma, nonsmall cell lung cancer, and bladder cancer [19]. All these studies suggested the promising clinical applications of tumor‐specific antigen‐derived vaccines in cancer immunotherapy.

Cancer immune subtyping classifies tumors into distinct subtypes according to the immune tumor microenvironment (TME), reflecting different responses to cancer immunotherapy [20, 21]. Cancer is not simply a group of tumor cells, but rather a heterogeneous collection of immune cells, stromal cells, extracellular matrix, and blood vessels [22, 23]. Thorsson et al. performed a comprehensive immunogenomic analysis of 33 cancer types from TCGA and identified six immune subtypes, including wound healing, IFN‐γ dominant, inflammatory, lymphocyte depleted, immunologically quiet, and TGF‐β dominant [24]. These immune subtypes had diverse prognoses, genetic, and immune modulatory alterations. For HCC, Xu's group found that a low‐risk HCC subtype showed increased immune cell infiltration and higher expression of checkpoints [25]. Zhuang et al. classified HCC into immunity high, immunity medium, and immunity low subtypes, which could aid patient selection for immunotherapy [26]. Another group identified four distinct immune subtypes with distinct prognoses and immune characteristics based on hundreds of immune signatures from HCC samples and detected dozens of potential immunological biomarkers for HCC [27]. These studies suggested that cancer immune subtyping may provide a novel strategy for future cancer therapeutic intervention. However, few studies have explored the immune signatures with tumor antigens and multiomics information in HCC.

In this study, we conducted a comprehensive analysis of potential tumor antigens and cancer immune subtyping from a multiomics perspective for HCC. We identified six potential tumor antigens that had a significant prognostic effect in HCC. Furthermore, two tumor antigens, ZNF831 and SYNE1, had positive correlations with antigen presenting cells. Afterward, a multiomics clustering was performed using the variably expressed tumor antigen genes of transcriptome, proteome, and phosphoproteome. In order to further explore the complex immune microenvironment in HCC, we identified 30 important immune‐related features and constructed four immune subtypes using consensus clustering. The four immune subtypes had distinct clinical and molecular characteristics. Our results provide novel insights into cancer immunity that probably aid the development of tumor antigen‐based vaccines and precision medicine of immunotherapy in HCC.

## 2 Materials and Methods

### 2.1 Data Source and Preprocessing

The clinical data, whole exon sequencing data, transcriptome, proteome, and phosphoproteome data of 159 paired HCC and adjacent tissues were obtained from the Clinical Proteomic Tumor Analysis Consortium (CPTAC)‐HCC project, which was previously published [27]. Clinical information including age, sex, Barcelona Clinic Liver Cancer (BCLC) stages, TNM stages, tumor thrombus, and survival data were obtained. Mutated genes may produce aberrant peptides, so we denoted the mutated genes with specific SNVs from WES data as tumor antigens. We defined the mutated genes with higher mutation frequency (> 5%) as common antigen genes. Three samples with no detected mutations were removed from the following analysis. The raw RNA counts of 17 571 genes were analyzed for differential expression analysis, and the upper‐quantile normalized RSEM counts with log2 transform were used for other analyses. The 6478 proteins and 26 418 phosphosites were normalized using the median centering method and log2 transformed.

### 2.2 Single Sample Gene Set Enrichment Analysis

We collected 22 immune cell gene signatures from a previously published study (Table S1) and evaluated the immune activities of these immune cells using single sample gene set enrichment analysis (ssGSEA) [28]. R package GSVA was used to perform the analysis.

### 2.3 Multiomics Clustering Using the Expression of Tumor Antigen Genes

We selected the top 5% variably expressed tumor antigen genes using the median absolute deviation method (MAD) for transcriptome, proteome, and phosphoproteome, respectively. Then min–max normalization was performed, resulting in a normalized multiomics data matrix. Afterward, we conducted a multiomics clustering using the normalized matrix and nonnegative matrix factorization (NMF) algorithm by R package (NMF), and two subtypes with distinct clinical and molecular features were identified.

### 2.4 Differential Analysis and Pathway Enrichment Analysis

Differential expression analysis between the two subtypes from multiomics clustering was performed using DESeq2 software and the cutoffs of FDR < 0.01 and foldchange > 2 were selected. Gene ontology (GO) enrichment analysis was conducted in the significantly increased or decreased genes using clusterProfiler R package and an FDR < 0.05 was considered as the cutoff of significantly regulated GOs.

### 2.5 Feature Selection by Random Forest Algorithm

In order to further explore the potential distinct immune environments of the two multiomics subtypes, we combined 252 immune‐related features including the RNA and protein expression of common tumor antigen genes (frequency > 5%), the activities of 22 immune cells, and 13 pathways associated with immune functions, such as EMT, cell cycle, nucleotide excision repair, and angiogenesis and so on (Table S2). Then the random forest algorithm was used to select immune‐related features which could best distinguish the two multiomics subtypes, and the mean decrease accuracy (MDA) analysis was used to identify the top 30 most important features. We also compared the predictive performance of the top 30 significant features and nonmetric multidimensional scaling analysis (NMDS) using all the combined features.

### 2.6 Identification of Immune Subtypes

Unsupervised consensus clustering was conducted using the identified top 30 immune related features to generate immune subtypes. The number of clusters was set to 2–10. We evaluated the average pairwise consensus matrix for consensus clusters, the consensus cumulative distribution function (CDF) curves, and the delta plot of the relative change in the area under CDF. The R package ConsensusClusterPlus was implemented for these analyses. To illustrate the distinct immune features among the immune subtypes, a heatmap was visualized. Moreover, principal component analysis (PCA) was performed using the top 30 features to demonstrate the robustness of the clustering.

### 2.7 Statistical Analysis

The statistical differences of continuous variables between more than two groups were compared by Kruskal–Wallis test. The statistical differences of categorical variables between groups were compared by Fisher's exact test or chi‐square test. Correlation between tumor antigen genes and antigen presenting cells was explored by Spearman correlation analysis. Kaplan–Meier curve and log‐rank test were used to visualize and compare the differences of overall survival (OS) and recurrence‐free survival (RFS) between groups. Univariable Cox proportional hazards model was implemented to explore the association between variables and survival. The *p* < 0.05 was considered statistically significant. All data preprocessing, statistical analyses, and data visualization were performed in R software v4.0.2.

## 3 Results

### 3.1 Correlation Between the Tumor Antigen Genes With Prognostic Effects and Antigen Presenting Cells

**FIGURE 1:**
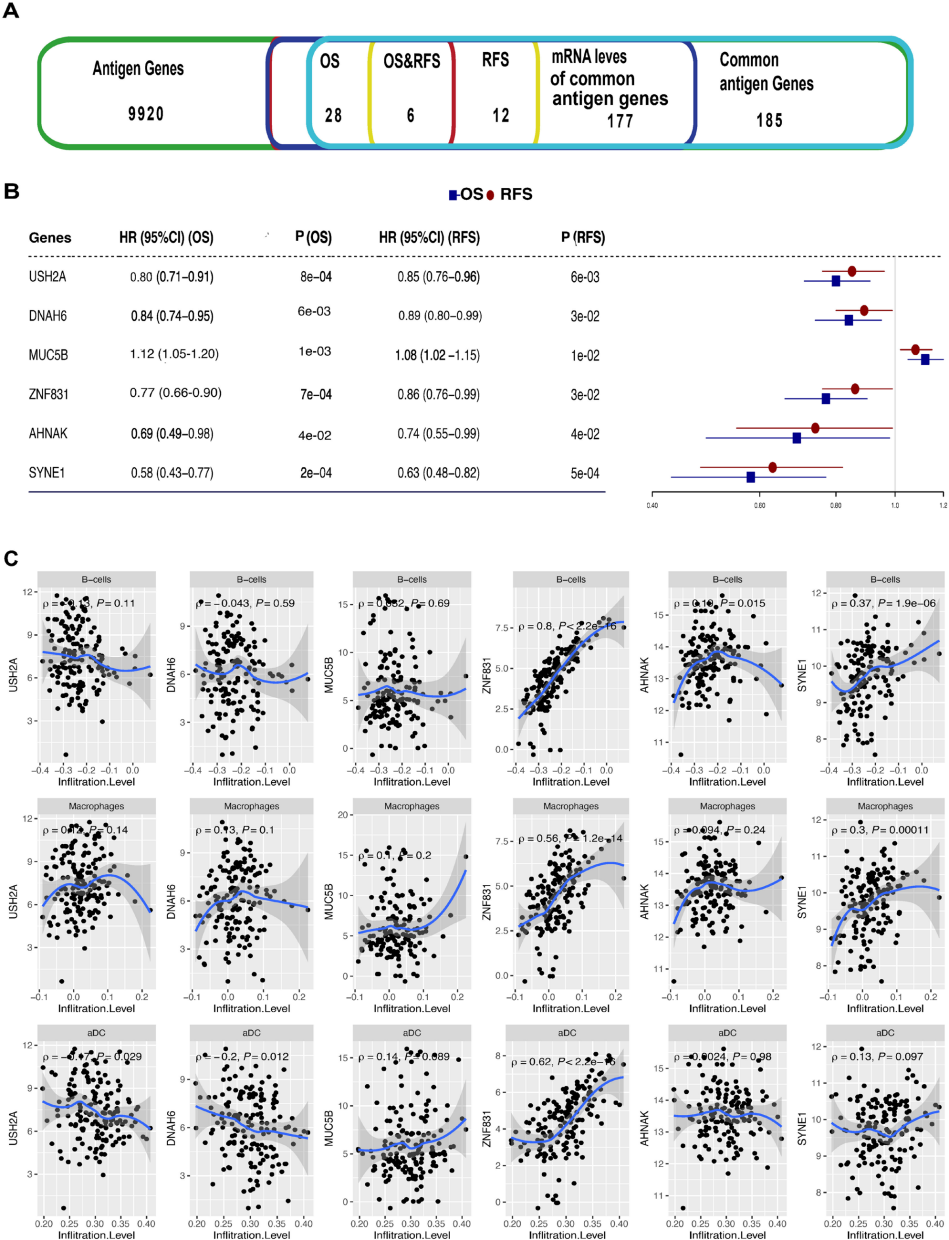
Identification of tumor antigen genes associated with HCC prognosis and antigen presenting cells. (A) Narrow‐down analysis of potential tumor antigen genes with significant association with OS and RFS. (B) The hazard ratios and *p* values of the six identified tumor antigen genes associated with both OS and RFS. (C) Scatter plot of the associations between the six identified tumor antigen genes and three antigen‐presenting cells.

In this study, a total of 159 overlapped samples with genome, transcriptome, proteome, and phosphoproteome information from the CPTAC‐HCC cohort were used in our analyses. The baseline clinical characteristics of the samples are summarized in Table S3. We denoted the mutated genes with specific SNVs from WES data as tumor antigens, which resulted in 9920 potential tumor antigen genes (Table S4). In order to screen tumor antigen genes that have prognostic effects and the potential to stimulate or inhibit the immune system, we conducted univariable Cox regression analysis for the 185 common tumor antigen genes (frequency > 5%). We found that the mRNA expression of 28 genes was closely associated with OS and 12 genes were associated with RFS (Figure 1A, Table S5). Six genes, including USH2A, DNAH6, MUC5B, ZNF831, AHNAK, and SYNE1, exerted prognostic associations with both OS and RFS, indicating their potential significant biological regulation effects in HCC (Figure 1B). We next explored the correlations between these six genes and antigen‐presenting cells, including B cells, macrophage cells, and dendritic cells (DCs). It showed that ZNF831 was positively correlated with all three of these immune cells (*p* < 0.05, Rho = 0.8, 0.56, 0.62, respectively, Figure 1C). Both the mRNA and protein expression of SYNE1 were positively correlated with B cells and macrophage cells (*p* < 0.05, Rho > 0.3, Figures 1C and S1A). Except for that, we observed that ZNF831 was also positively associated with CD4+ T cells and CD8+ T cells (Figure S1B). These results suggest that mutations in ZNF831 and SYNE1 may produce tumor antigens that have the potential to be processed and presented by the immune cells and can be potential candidate biomarkers aiding the development of HCC mRNA vaccines.

### 3.2 Multiomics Clustering of HCC Using the Expression of Tumor Antigen Genes

**FIGURE 2:**
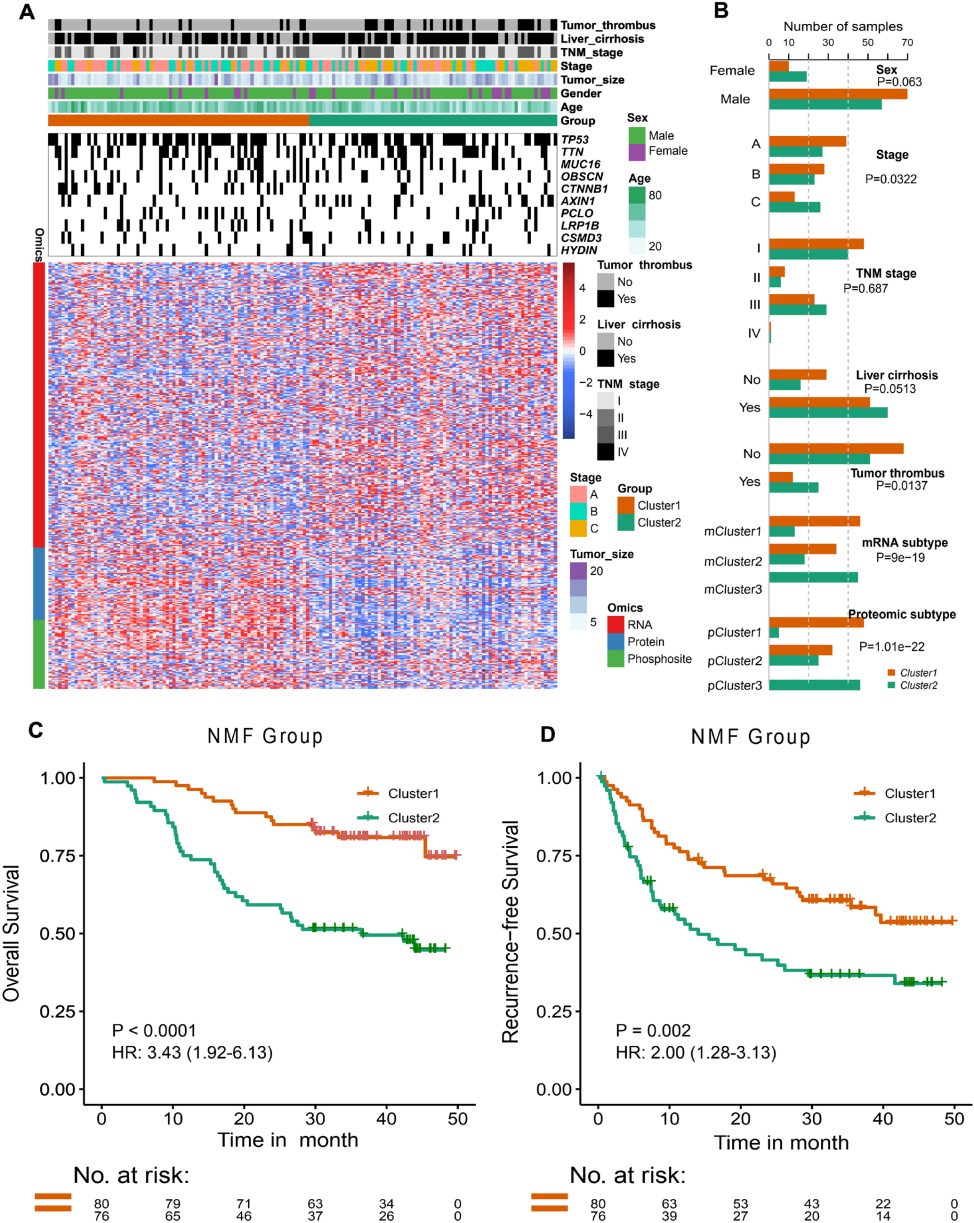
Multiomics stratification of HCC samples using the top variably expressed tumor antigen genes and the clinicopathologic correlations. (A) Multiomics clustering based on the top 5% most variable tumor antigen genes for transcriptome, proteome and phosphoproteome using NMF algorithm. (B) The associations between the subtypes identified from multiomics clustering and clinicopathologic factors. (C) Kaplan–Meier curves for overall survival (OS) between multiomics subtypes. (D) Kaplan–Meier curves for recurrence free survival (RFS) between multiomics subtypes.

Depending on the tremendous multiomics data, we performed a multiomics clustering based on the top 5% variably expressed antigen genes for transcriptome, proteome, and phosphoproteome, respectively. As shown in Figure 2A, two subtypes were generated using the multiomics datasets. We found that subtype 1 was characterized by the enrichment of samples with low BCLC stages and without tumor thrombus (*p* < 0.05). Compared with transcriptome and proteome clustering from the previous study [27], subtype 1 was enriched in transcriptome cluster 1 and proteome cluster 1, whereas subtype 2 had a higher proportion of transcriptome cluster 3 and proteome cluster 3 (*p* < 0.05, Figure 2B). Consistent with the previous findings that transcriptome cluster 1 and proteome cluster 1 had the best prognosis, subtype 1 in our study showed a remarkably better OS and RFS than subtype 2 (OS, *p* < 0.0001, HR: 3.43, 95% CI: 1.92–6.13, Figure 2C; RFS, *p* = 0.002, HR: 2.00, 95% CI: 1.28–3.13, Figure 2D). In order to uncover the distinct biological functions between these two subtypes, we conducted differential expression and GO enrichment analysis. There were 77 and 102 genes significantly increased in subtype 1 and subtype 2, respectively. It revealed that the increased genes in subtype 1 were enriched in metabolic functions of fatty acid, steroid, and drugs, whereas the increased genes in subtype 2 were related to extracellular matrix organization and leukocyte activities (Figure S2 and Table S6). These results suggested that tumor antigen‐based multiomics clustering could provide novel clinical and molecular diversities across HCC patients.

### 3.3 Immune Feature Selection and Identification of Potential Immune Subtypes of HCC

**FIGURE 3:**
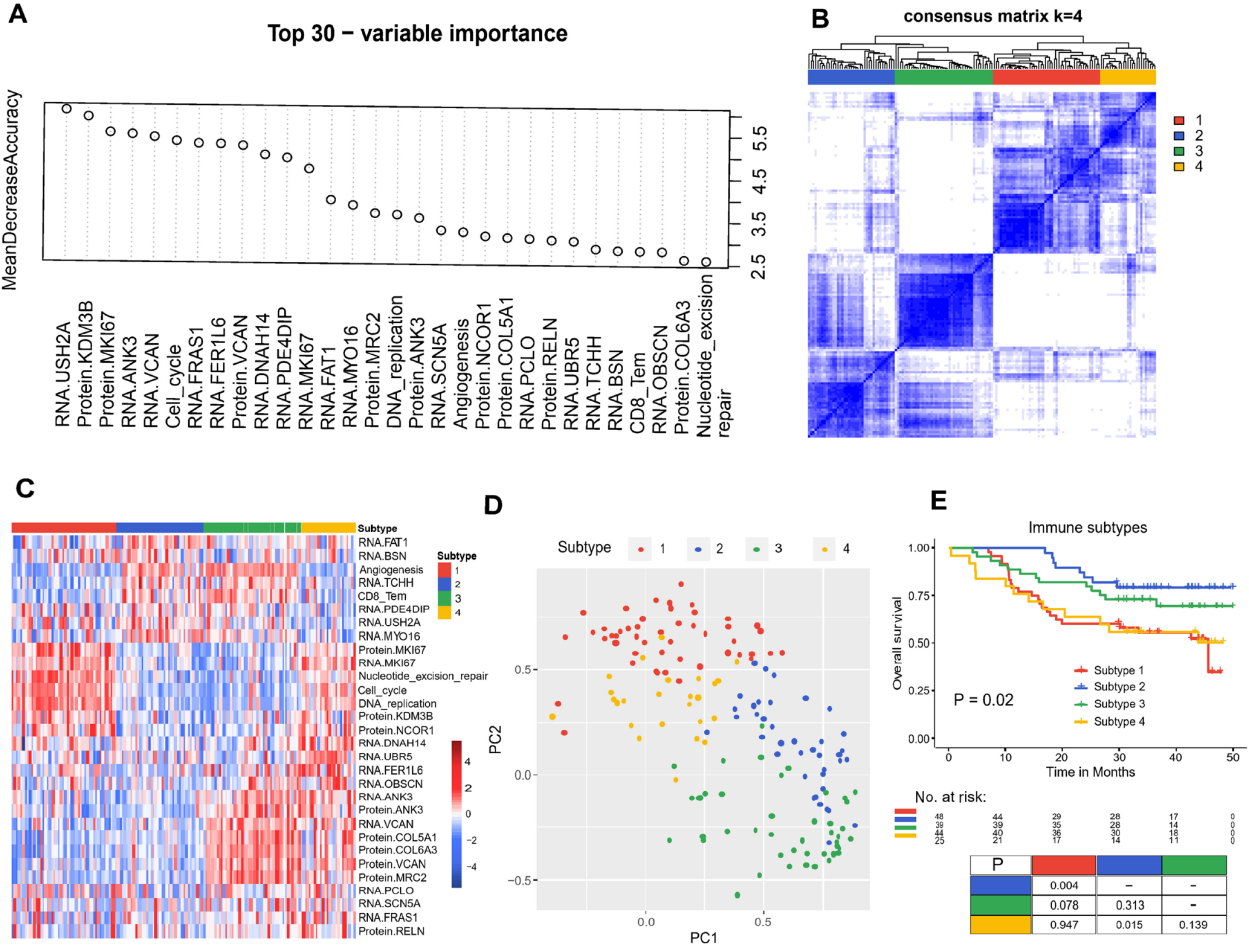
Feature selection and immune subtyping using the top 30 immune related features. (A) The top 30 important features identified by mean decrease accuracy algorithm (MDA). (B) Heatmap depicting the consensus matrix when *k* = 4 was selected. (C) Heatmap of immune subtypes with distinct immune related characteristics. (D) Principal component analysis (PCA) for HCC patients using the top 30 immune related features. Colors represented the four immune subtypes. (E) Kaplan–Meier curves for overall survival between the immune subtypes. *p* values of pairwise comparisons of survival differences were listed in the bottom table.

To further elucidate the complex biological relationships between the identified tumor antigen‐based subtypes and immune features in HCC, we then combined the RNA and protein expression of the common tumor antigen genes, the levels of 22 tumor‐infiltrating immune cells, and 13 activity scores of immune‐related pathways, and then conducted feature selection analyses for these two multiomics subtypes using random forest. According to the MDA algorithm, the top 30 immune‐related features were selected, including CD8_Tem, cell cycle, angiogenesis, and the RNA and protein expression of several tumor antigen genes (Figures 3A and S3A). The samples predicted from the top 30 immune‐related features could be excellently distinguished by the NMDS method using all the combined features (Figure S3B). These results suggest that these features may play a significant role in immune regulation of HCC.

Cancer immune subtyping has been a hot area of research, and it may provide important clinical guidance for immunotherapy aiding precision medicine. We next performed an in‐depth consensus clustering analysis using the selected 30 immune features. According to the consensus matrix, consensus CDF plot, and delta area plot (Figures 3B and S3C,D), four immune subtypes were generated with distinct molecular functions (Figure 3C). Specifically, immune subtype 1 was featured by the increased proliferative pathways, such as DNA replication, the cell cycle, and nucleotide excision repair. On the contrary, subtypes 2 and 3, with low proliferative pathways, were characterized by high angiogenesis, CD8_Tem, and the RNA expression of some potential tumor antigen genes, such as TCHH, PDE4DIP, and MYO16. Except for that, subtype 3 exhibited additional high protein expression of potential tumor antigen genes, including ANK3, COL5A1, COL6A3, VCAN, and MRC2. Subtype 4, which was different from the other three subtypes, showed decreased angiogenesis and CD8_Tem and higher levels of other selected features. The PCA analysis using the top 30 immune features also showed robust discrimination between the four immune subtypes (Figure 3D). We also explored the survival difference between the four immune subtypes, and as shown in Figure 3E, immune subtypes 2 and 3 displayed better prognosis compared with subtypes 1 and 4.

### 3.4 Association Analysis of the Immune Subtypes With Clinical and Mutational Characteristics

**FIGURE 4:**
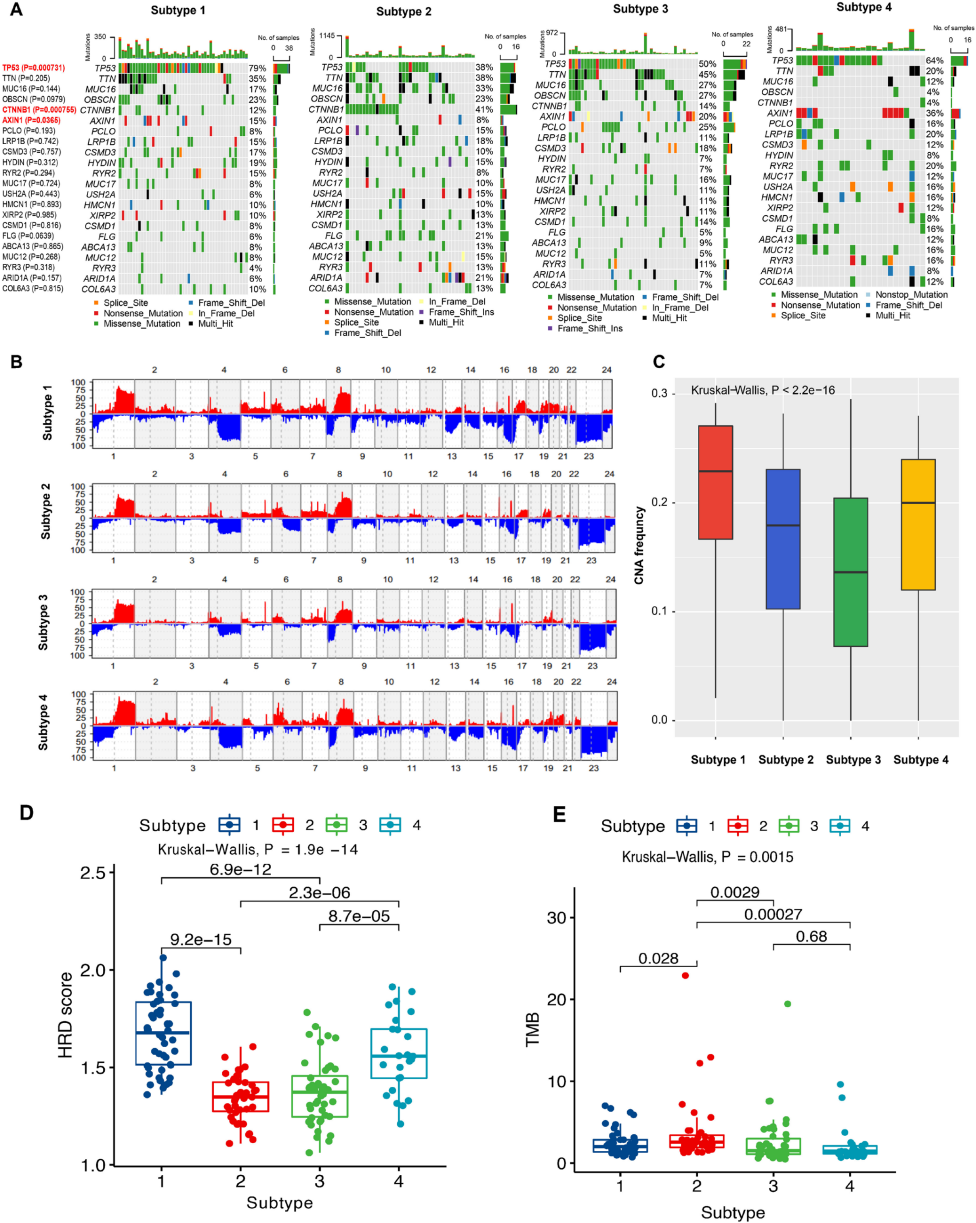
The mutational characteristics associated the immune subtypes. (A) The mutation landscape of the top mutated genes (frequency > 10%) between the four immune subtypes. (B) The copy number alteration (CNA) landscape between the four immune subtypes. (C) The copy number alteration (CNA) frequency between the four immune subtypes. (D) The homologous recombination deficiency (HRD) scores between the four immune subtypes. (E) The tumor mutation burden (TMB) scores between the four immune subtypes.

We then compared the associations between the four immune subtypes and clinical features. The immune subtype 3 was significantly enriched in low BCLC stage (A), whereas patients in immune subtype 4 had a higher proportion of stage C (*p* < 0.05, fisher exact test, Figure S4). Except for that, patients with tumor thrombus were also enriched in immune subtype 4. These results revealed that patients with different clinical settings might have distinct immune characteristics. The mutation landscape plays significant roles in regulating cancer immunity. Therefore, we investigated whether the top mutated genes (frequency > 10%) had a preference for the four immune subtypes. TP53 was significantly mutated in subtype 1, whereas the mutations in CTNNB1 and AXIN1 were prominently enriched in immune subtypes 2 and 4, respectively (*p* < 0.05, fisher exact test, Figure 4A). As for the copy number alterations (CNA), we found that immune subtypes 1 and 4 had higher CNA frequencies compared with subtypes 2 and 3 (*p* < 0.05, Kruskal–Wallis test, Figure 4B,C). Previous studies demonstrated that homologous recombination deficiency (HRD) scores and tumor mutation burden (TMB) scores are important biomarkers in cancer immunotherapy [29, 30]. We found that subtype 1 had the highest HRD score among the four immune subtypes (*p* < 0.05, Figure 4D), which was consistent with the enrichments of TP53 mutations and proliferative pathways. The TMB score showed the highest levels in subtype 2 (Figure 4E), suggesting the potential effectiveness of PD1/PDL‐1 immunotherapy in this subtype.

### 3.5 Association Analysis of the Immune Subtypes With ICPs and Immunogenic Cell Death (ICD) Modulators

**FIGURE 5:**
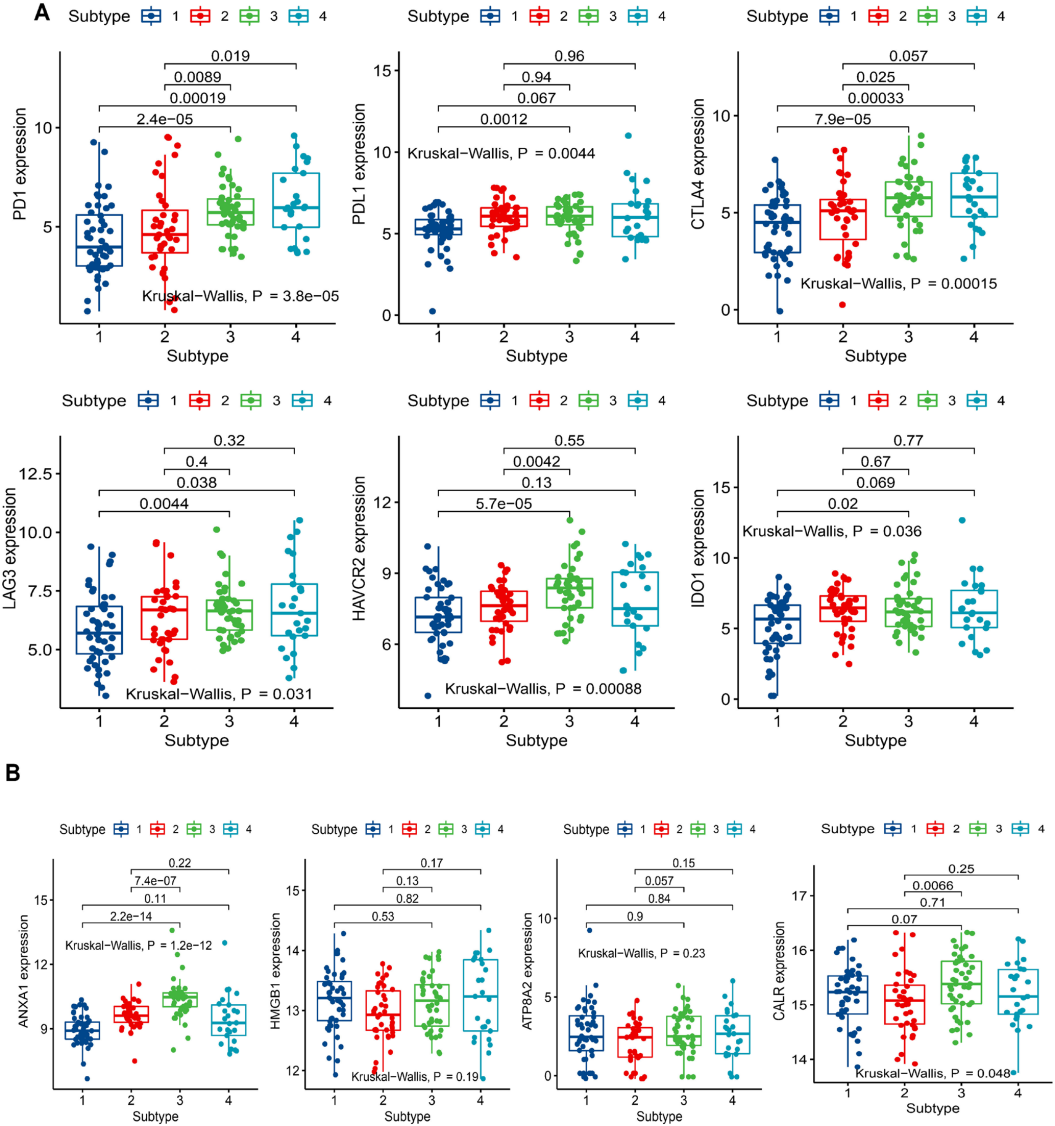
The expression of immune checkpoints (ICPs) and immunogenic cell death (ICD) modulators associated with immune subtypes. (A) The expression of immune checkpoints (ICPs) between the four immune subtypes. (B) The expression of immunogenic cell death (ICD) modulators between the four immune subtypes.

ICP blockades have become the most effective antitumor therapy in multiple cancer types. ICD modulators are molecules secreted from the ICD process which could activate both the innate and adaptive immune responses [31]. All these two types of genes have significant regulatory effects for cancer immunotherapy. Therefore, we investigated the expression of six proverbial ICPs and four ICD modulators among the four immune subtypes. PD‐1 and CTLA‐4 showed higher expression in subtypes 3 and 4 than in subtypes 1 and 2, whereas HAVCR2 was highly expressed in subtype 3 (*p* < 0.05, Figure 5A). Furthermore, PD‐L1, LAG3, and IDO1 exhibited the lowest expression in subtype 1 compared with the other three subtypes (*p* < 0.05, Figure 5A). The distinct expression levels of ICPs among the four subtypes suggest that personalized immunotherapy targeting particular molecules needs to be considered for diverse immune subtypes. For the four ICD modulators, we found that both ANXA1 and CALR showed the highest expression in immune subtype 3 (*p* < 0.05, Figure 5B). According to previous studies, cancer vaccines generated from tumor antigens had increased effectiveness with the upregulation of these ICD modulators [32, 33]. Patients from immune subtype 3 may achieve superior clinical benefits for tumor antigen‐based cancer vaccine development.

## 4 Discussion

HCC is one of the most deadly cancers with limited therapeutic interventions [1]. Although PD‐1/PD‐L1 blockade has achieved revolutionary success in cancer treatment over the last decade, only 15%–20% of HCC patients showed durable clinical benefits and objective remissions, and novel therapeutic strategies are needed to improve the clinical management [34, 35, 36]. In this study, we integrated potential tumor antigens analysis and cancer immune subtyping from a multiomics perspective in HCC. We identified two potential tumor antigen genes with significant prognosis and associations of antigen processing cells. In addition, four immune subtypes were constructed which showed distinct clinical and molecular characteristics. Our results provide novel insights into the development of tumor antigen‐based cancer vaccines and precision immune oncology in HCC.

Tumor specific antigens are variant peptides which can be recognized by immune cells and act as ideal targets to increase antitumor immunity [15, 16]. In this study, we identified 185 common tumor antigen genes with high frequencies (> 5%) in 159 HCC patients. Among them, six tumor antigen genes showed significant prognostic effects for OS and RFS, and two genes, ZNF831 and SYNE1, also exhibited positive associations with antigen presenting cells including B cells, macrophage cells, or DCs. ZNF831 is a zinc finger protein related to nucleic acid binding. Jiang et al. discovered that ZNF831 was an important immunology regulon associated with survival in 11 cancer types, and Chen et al. found ZNF831 was a signature gene of leukocyte fraction [37, 38]. SYNE1 encodes a spectrin repeat containing protein and plays vital roles in cytoskeletal function, DNA damage response, and genome instability [39]. A recently published study suggested that SYNE1 mutation was associated with a higher TMB and a worse survival in clear cell renal cell carcinoma and may enhance the response to PD‐1/PD‐L1 blockade therapy [40]. All these results implied that ZNF831 and SYNE1 may be promising candidates for tumor antigen based cancer vaccine development in HCC.

HCC is a highly heterogeneous disease, and molecular and immune subtyping provides more accurate classification of cancer, adding the ultimate goal of precision medicine. We first conducted a multiomics clustering using the variably expressed tumor antigen genes. Two subtypes with distinct metabolism, extracellular matrix organization, and leukocyte activities were constructed. In order to further explore the complex immune microenvironment of HCC, we first performed a feature selection analysis using 252 combined immune‐related features and the random forest algorithm. Four immune subtypes were clustered using the top 30 important features. We found that immune subtype 1 was associated with TP53 mutation and proliferative features, such as the cell cycle and DNA replication. Inhibitors of cyclin‐dependent kinases (CDKs) may represent effective therapeutic opportunities for these patients [41, 42, 43]. The immune subtype 2 had a close association with CTNNB1 mutation, angiogenesis, CD8 Tem, and high TMB, suggesting that a combination of PD‐1/PD‐L1 blockade and antiangiogenesis therapy may provide better clinical benefits for this population [44, 45]. Immune subtype 3 had the highest expression of ICD modulators, such as ANXA1 and CALR, and several potential tumor antigen genes (ANK3, VCAN, and MRC2 etc.). Tumor antigen‐based cancer vaccine therapy may be a promising clinical treatment for this subtype [46, 47]. Except for that, we found the expression of two ICPs, PD‐1 and CTLA‐4, was higher in immune subtype 4, implying that antibodies against CTLA‐4 and PD‐1 may be more suitable for this subset of patients [48]. Compared with previously published results in this field, our study provided a novel personalized treatment regimen for the particular immune HCC subtype based on integrating multiomics information of potential tumor antigen genes, which may benefit clinical practice in future [25, 26, 49].

Although novel tumor antigen analysis and immune subtyping were provided in the current study, limitations should not be ignored. This study was a retrospective study, which may limit the strength of evidence. First, current studies implied that different etiologies of HCC have distinct clinical, genetic and immune characteristics [50, 51]. HBV‐related HCC is poorly differentiated with higher serum AFP levels and favors the proliferation subtype with PI3K‐AKT–mTOR activation [50]. On the contrary, the HCV and alcohol‐related HCC exhibit a nonproliferation phenotype with moderately‐to‐well differentiation, lower serum AFP levels, and higher CTNNB1 and TERT promoter mutation. Llovet et al. also suggested that the microenvironmental features and immune response differed between different etiologies related to HCC [51]. Exploration of the molecular and immune features of other etiologies of HCC still needs further exploration. Second, independent validation of the immune subtypes using larger, multicenter cohorts would strongly strengthen the current conclusions. Other publicly available cohorts with such tremendous multidimensional data are urgently needed, especially for proteome and phosphoproteome data. Third, the two identified tumor antigen genes, ZNF831 and SYNE1, which have the potential to aid the development of cancer vaccines, should be viewed as hypothesis generating rather than conclusions, because the causal effects of the correlations cannot be determined in the current study. Future work of cell line and clinical trial evaluation is warranted to further investigate and validate these biological and treatment hypotheses.

In conclusion, we identified two potential tumor antigen genes, ZNF831 and SYNE1, which showed significant prognostic effects and positive correlations with antigen presenting cells. We also performed tumor antigen genes‐based multiomics subtyping and detailed immune subtyping. These results provide novel insights about the development of cancer vaccines and precision medicine in immune oncology in HCC.

## Author Contributions

**Chunming Wang:** data curation (lead), formal analysis (lead), visualization (lead). **Lei Cai:** data curation (equal), formal analysis (equal), visualization (supporting). **Qingyu Xie:** formal analysis (equal), investigation (equal), validation (equal). **Cheng Zhang:** formal analysis (equal), methodology (equal), visualization (supporting). **Xuefang Chen:** formal analysis (equal), investigation (supporting). **Peng Cui:** formal analysis (equal), visualization (equal). **Guoqiang Wang:** formal analysis (supporting), investigation (equal), resources (equal). **Shangli Cai:** project administration (lead), supervision (equal). **Yusheng Han:** funding acquisition (equal), writing – review and editing (equal). **Kaihang Zhong:** formal analysis (supporting), methodology (equal). **Guolin He:** data curation (equal). **Shunjun Fu:** data curation (equal). **Yuyan Xu:** data curation (equal), methodology (supporting). **Junming He:** conceptualization (equal), investigation (supporting), writing – review and editing (lead). **Mingxin Pan:** conceptualization (lead), funding acquisition (lead), supervision (equal), writing – original draft (lead).

## Ethics Statement

The authors have nothing to report.

## Consent

The authors have nothing to report.

## Conflicts of Interest

Peng Cui, Guoqiang Wang, Shangli Cai, and Yusheng Han are employees of Burning Rock Biotech. The other authors declare no conflicts of interest.

## Supporting information


**Figure S1:** The Spearman correlations between the identified prognostic tumor antigen genes and the infiltration of representative immune cells.
**Figure S2:** The gene ontology enrichment analysis for the subtype 1 compared with subtype2 from multiomics clustering.
**Figure S3:** Feature selection and immune subtyping using the top 30 immune related features.
**Figure S4:** The clinicopathologic factors associated with the four immune subtypes. Numbers were calculated with ‐log10 (*p* value). Fisher exact test was adopted and significant associations were labeled with asterisks.

**Data S1:** Supplementary Tables.

## Data Availability

The datasets for this study are obtained from the CPTAC‐HCC project and can be viewed in NODE (https://www.biosino.org/node) by pasting the accession (OEP000321).
