# Supplementary material for: Identification of Tumor Antigens and Immune Subtypes in Hepatocellular Carcinoma From a Multiomics Perspective
Source: Cancer Rep (Hoboken). 2025 Sep 15;8(9):e70300. doi: 10.1002/cnr2.70300 (PMC12434600; doi:10.1002/cnr2.70300)
Supplement: Supplementary file 1 — Figure S1: The Spearman correlations between the identified prognostic tumor antigen genes and the infiltration of representative immune cells. Figure S2: The gene ontology enrichment analysis for the subtype 1 compared with subtype2 from multiomics clustering. Figure S3: Feature selection and immune subtyping using the top 30 immune related features. Figure S4: The clinicopathologic factors associated with the four immune subtypes. Numbers were calculated with ‐log10 (p value). Fisher exact test was adopted and significant associations were labeled with asterisks. [file CNR2-8-e70300-s001.docx]

**
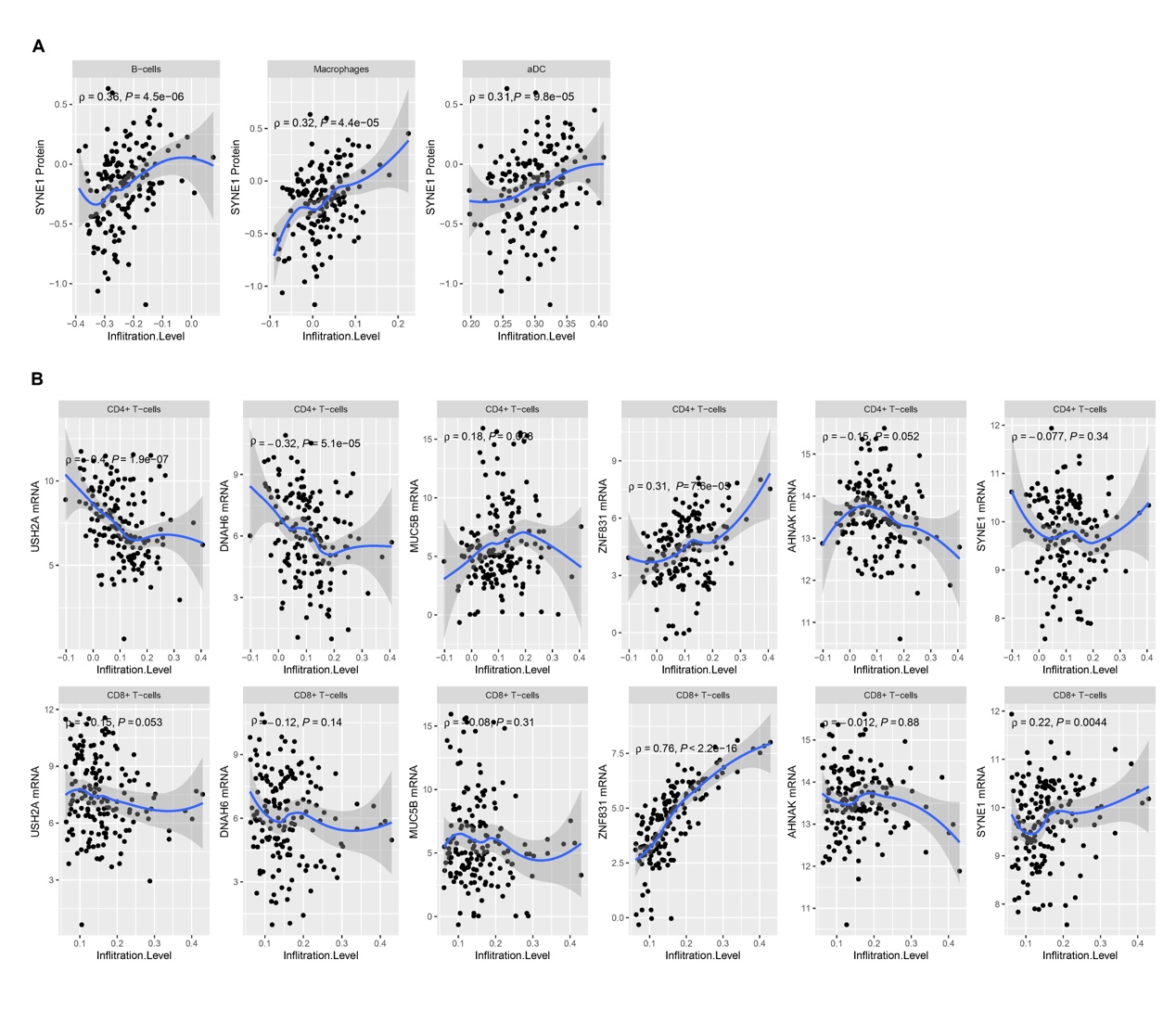
**

**Figure S1. The Spearman correlations between the identified prognostic tumor antigen genes and the infiltration of representative immune cells.**

1. The Spearman correlations between the protein expression of SYNE1 and the infiltration of three antigen presenting cells.
2. The Spearman correlations between the mRNA expression of the 6 identified prognostic tumor antigen genes and the infiltration of CD4+ T cells and CD8+ T cells.


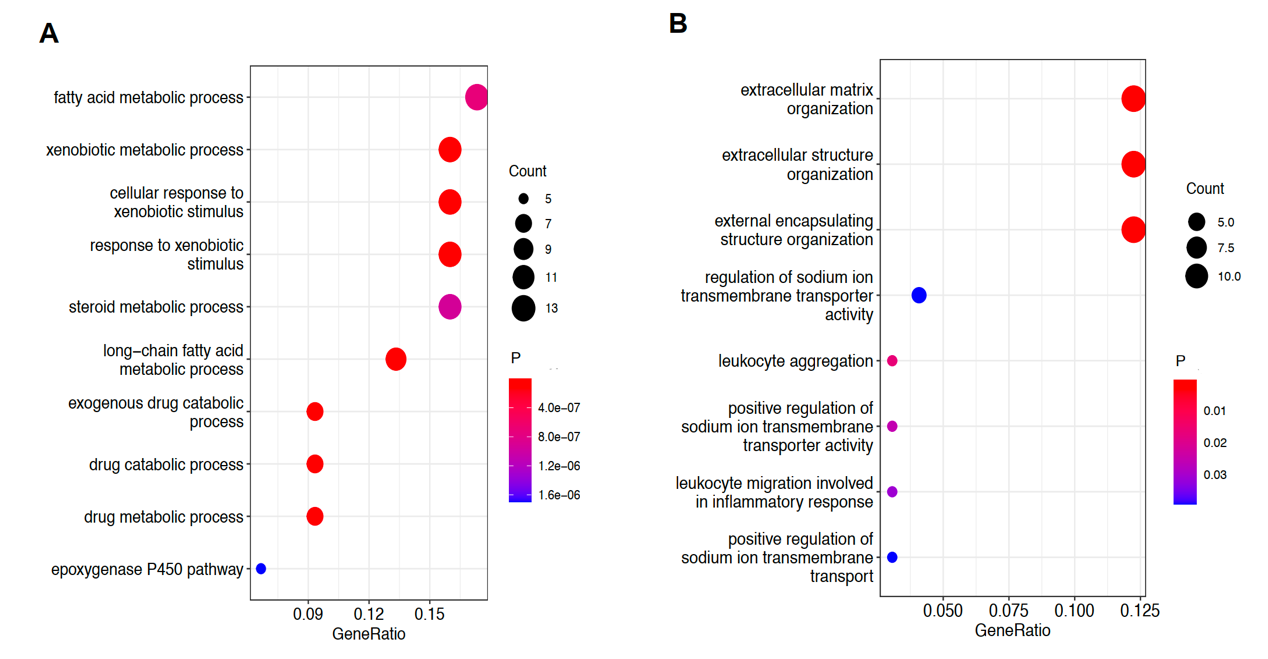


**Figure S2. The gene ontology enrichment analysis for the subtype 1 compared with subtype2 from multi-omics clustering.**

1. The up-regulated gene ontologies in the subtype 1 compared with subtype 2 (FDR < 0.05).
2. The down-regulated gene ontologies in the subtype 1 compared with subtype 2 (FDR < 0.05).


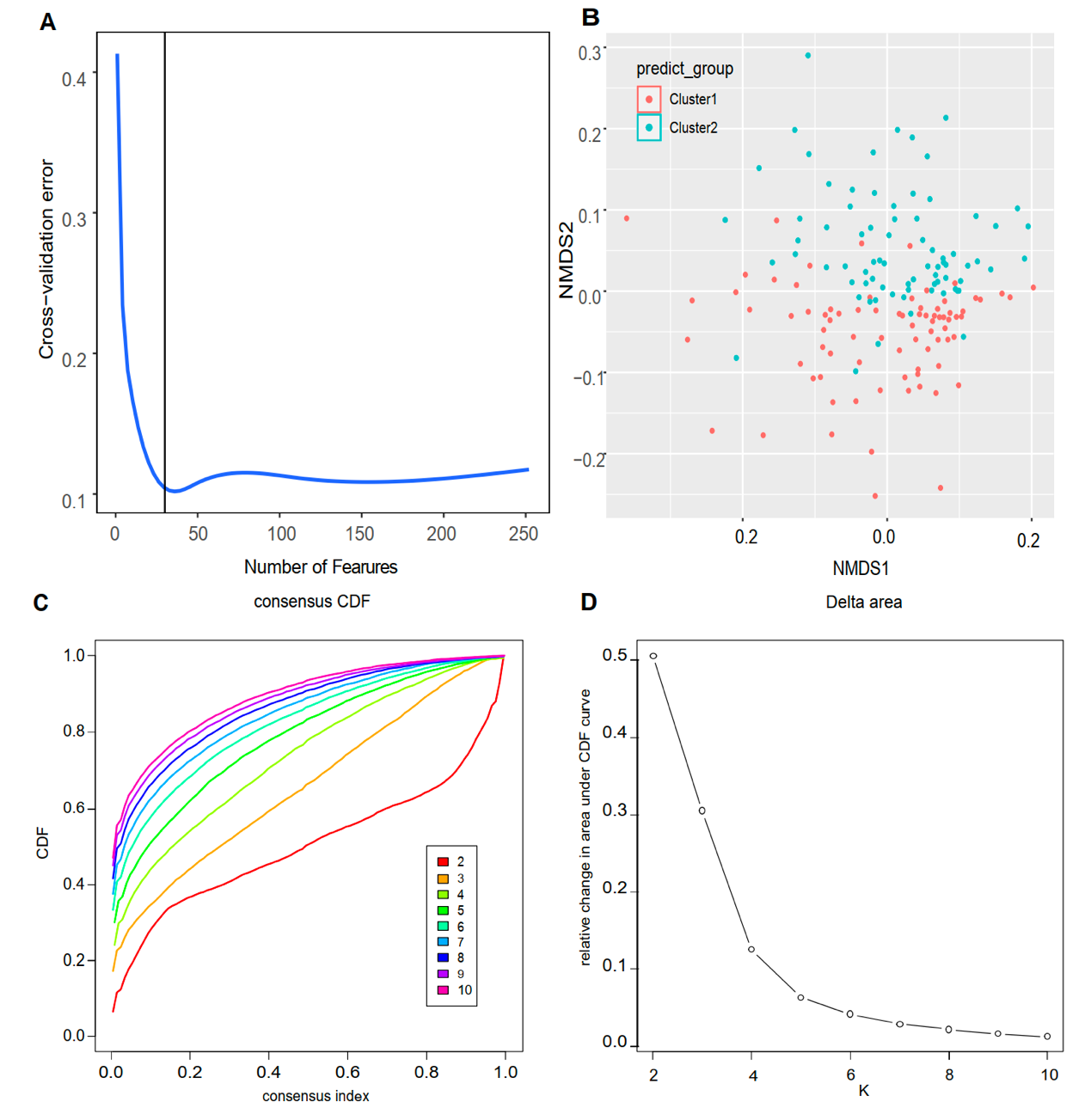


**Figure S3. Feature selection and immune subtyping using the top 30 immune related features.**

1. The cross-validation error curve of random forest using different number of features.
2. The nonmetric multidimensional scaling (NMDS) plot using 252 immune related features. The red and blue colors indicate the predicted multi-omics subtypes using the top 30 important features and random forest.
3. The consensus ﻿cumulative distribution function (CDF) plot was visualized from k = 2 to 10.
4. The delta area plot was illustrated from k = 2 to 10.


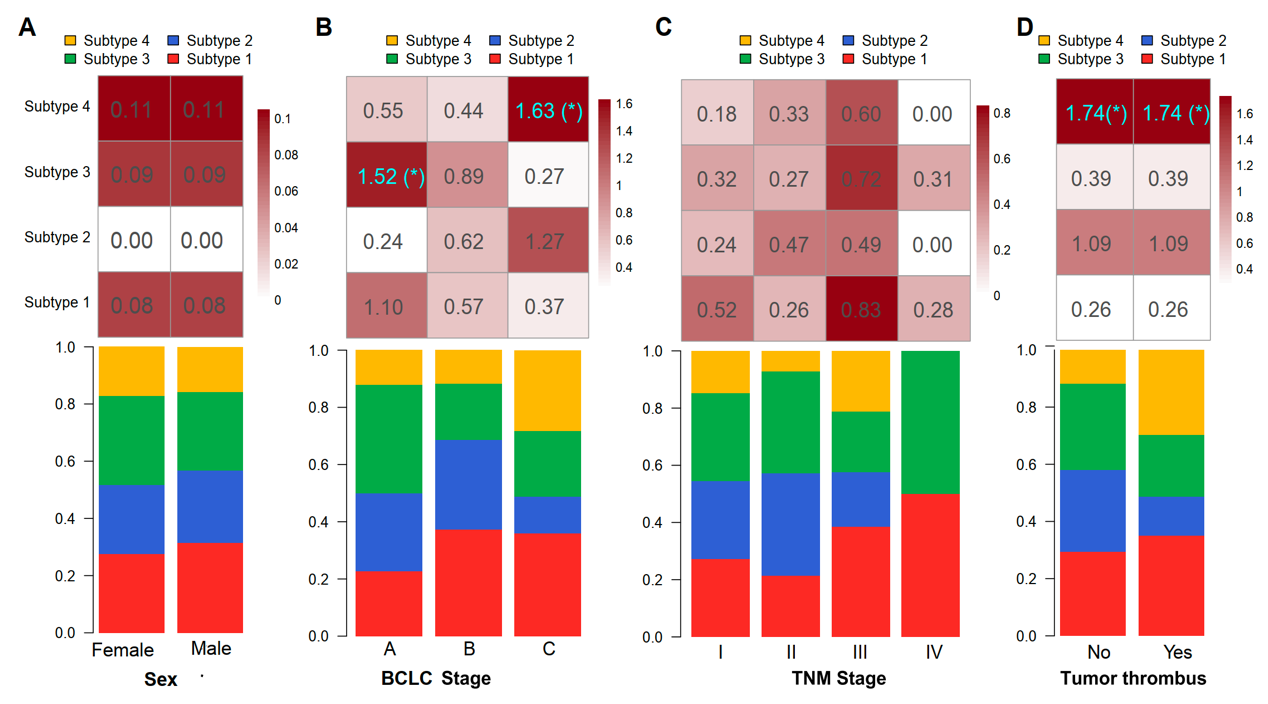


**Figure S4. The clinicopathologic factors associated with the four immune subtypes.** Numbers were calculated with -log10 (P value). Fisher exact test was adopted and significant associations were labeled with asterisks.
